# Patient Characteristics Associated With Non-acceptance of Intermittently Scanned Continuous Glucose Monitoring (isCGM): A Retrospective Single-Center Study

**DOI:** 10.7759/cureus.107766

**Published:** 2026-04-26

**Authors:** Shunsuke Hayashi, Kiminori Konaka, Risako Hamada

**Affiliations:** 1 Department of Hematology and Diabetes/Endocrinology, Japan Community Health Care Organization (JCHO) Tokuyama Central Hospital, Shunan, JPN; 2 Department of Diabetology, Endocrinology and Metabolism, Yamaguchi Prefectural Grand Medical Center, Hofu, JPN

**Keywords:** acceptance of iscgm, diabetes, logistic regression analysis, penalized model, treatment intensity

## Abstract

Objective

Intermittently scanned continuous glucose monitoring (isCGM) improves glycemic outcomes in patients with diabetes; however, some patients decline its use even when it is recommended by healthcare providers. We investigated patient characteristics associated with acceptance of isCGM in individuals with type 2 diabetes treated with insulin.

Methods

This single-center, retrospective study included outpatients with type 2 diabetes who were receiving insulin therapy and were advised to use isCGM by a single physician between June 2022 and December 2024. Patients with impaired decision-making capacity, clear reasons for declining device attachment (such as dermatological disease), definite type 1 diabetes, or gestational diabetes were excluded. The primary outcome was non-acceptance of isCGM after physician recommendation. Fisher's exact test and the Wilcoxon test were performed to compare the isCGM acceptance group with the non-acceptance group.

Univariate and multivariable logistic regression analyses were performed using acceptance or non-acceptance of isCGM as the dependent variable. To address potential overfitting and correlations among predictors, elastic net penalized logistic regression with 10-fold cross-validation was performed. Lambda (λ) is the tuning parameter that controls the strength of the penalty term in the loss function. The optimal λ value was selected for model regularization, and predictors with non-zero coefficients at that value were selected. Bidirectional stepwise selection was used as a sensitivity analysis.

Results

A total of 142 patients were included, of whom 98 accepted isCGM and 44 did not. Compared to the acceptance group, the non-acceptance group had a higher total insulin dose, longer duration of insulin use, and higher body mass index (BMI). In univariate logistic regression, log-transformed total daily insulin dose, log-transformed duration of insulin use, and BMI were associated with lower odds of accepting isCGM, whereas no variable remained statistically significant in the multivariable model. In elastic net analysis, BMI, log-transformed total daily insulin dose, log-transformed duration of insulin use, and log-transformed HbA1c were selected. The area under the curve was 0.63, suggesting modest discriminative ability. Repeated elastic net analyses showed high selection frequencies for BMI, total insulin dose, duration of insulin use, and HbA1c. Age was not meaningfully associated with acceptance.

Conclusion

Treatment intensity, reflected by a higher insulin dose, longer duration of insulin use, and a higher number of insulin injections, may be associated with declining isCGM. Higher HbA1c may be associated with willingness to accept isCGM, whereas older age alone may not be a major barrier. These exploratory findings require confirmation in larger prospective studies.

## Introduction

Intermittently scanned continuous glucose monitoring (isCGM) using the FreeStyle Libre system (Abbott Diabetes Care, Alameda, California, United States) is useful for glycemic control in patients with diabetes. Using isCGM, patients with diabetes can simply scan a sensor on their body surface to determine their up-to-date glucose levels at any given time. The use of isCGM reportedly improves hemoglobin A1c (HbA1c) levels and quality of life while reducing the frequency of hypoglycemia in patients with type 1 and type 2 diabetes, as shown in several clinical trials [1-3].

Nevertheless, some patients do not accept the use of isCGM. Moreover, it remains unclear which patients will accept or not accept the use of isCGM when recommended by healthcare providers. Healthcare professionals may find that some patients do not accept isCGM even in the absence of clear reasons, such as dermatological conditions (e.g., bullous pemphigoid or skin irritation). In Japan, isCGM is covered by insurance for all patients with diabetes who are receiving insulin therapy. Based on an exchange rate of 1 USD=147 JPY, the additional out-of-pocket cost for isCGM under the standard 30% copayment was $25.50. This was higher than the cost of one self-monitoring of blood glucose (SMBG) test per day ($9.50) but lower than that of four SMBG tests per day ($30.40). In many cases, the reasons why patients do not accept continuous glucose monitoring (CGM) when recommended remain unknown, and patient characteristics associated with acceptance or non-acceptance remain unclear.

Barriers to CGM use among patients and healthcare professionals, particularly in type 1 diabetes and issues related to adherence, have been explored [4,5]. Moreover, obstacles to CGM adoption from both patient and healthcare provider perspectives have been examined [6]. However, few studies have investigated the acceptance or non-acceptance of isCGM in populations with predominantly type 2 diabetes.

This study was not designed to formulate and test hypotheses; rather, it was conducted as an exploratory, hypothesis-generating study to generate testable hypotheses in areas where the relationships remain unclear. The purpose of this study was to investigate whether patients with type 2 diabetes accepted the recommendation to use isCGM and, by analyzing background factors, to identify the factors associated with acceptance or non-acceptance. Identifying the background characteristics and tendencies of patients who do not accept isCGM may help healthcare providers develop better strategies.

Part of this report was presented at the 62nd Annual Meeting of the Chugoku-Shikoku Branch of the Japan Diabetes Society on December 7, 2024.

## Materials and methods

Study design

This single-center, retrospective study included patients under the care of a single physician.The study population comprised patients with type 2 diabetes mellitus receiving outpatient care at our hospital, Japan Community Health Care Organization (JCHO) Tokuyama Central Hospital, located in Shunan, Japan. Data were collected retrospectively from the electronic medical records. The study received approval from the Ethics Committee of Tokuyama Central Hospital (approval number: K486-20250205).

Participants

In Japan, since April 2022, patients undergoing insulin therapy at least once daily have been eligible to use isCGM under the national health insurance system. Our hospital is a large community-based regional core hospital. Between June 2022 and December 2024, 354 patients had records of receiving insulin at our hospital. Physician A recommended isCGM at least once to nearly all outpatients receiving at least one daily insulin injection. Because the process by which other physicians recommended isCGM was unclear, the analysis was restricted to patients to whom Physician A had recommended isCGM in the outpatient setting.

Eligible patients were those who were able to communicate without difficulty during outpatient visits and were judged capable of making their own medical decisions. Patients who were unable to decide for themselves because of apparent dementia or other reasons, or who required family support to make decisions, were excluded. A total of 169 patients were enrolled. Next, patients with a clear reason for non-accepting device attachment, such as dermatological disease, were excluded. Patients with definite type 1 diabetes or gestational diabetes were also excluded. Consequently, 142 patients were included in the final analysis. These patients were considered capable of independently providing informed consent for treatment, and the process by which isCGM was recommended to them was regarded as consistent.

isCGM recommendation process 

Since 2022, Dr. A has been in charge of our outpatient clinic and responsible for patient care. The consultations were conducted in private rooms, with an administrative staff member present, but without the involvement of nursing or other medical staff. Dr. A explained the isCGM device to all patients administering insulin injections who visited for routine outpatient care while showing them the device itself. He emphasized the advantages of device use, including the ability to visualize blood glucose levels graphically, to understand nocturnal blood glucose levels that patients cannot measure on their own, to detect unexpected hypoglycemia or hyperglycemia, and to minimize the need for fingertip blood sampling. As disadvantages, the need to continuously wear the sensor for two weeks and potential measurement errors were highlighted. Nevertheless, he explained that the benefits outweighed the disadvantages and recommended using the device. When patients had questions, Dr. A explained the cost implications.

In these consultations, Dr. A emphasized a patient-centered approach and informed consent, allowing patients to freely decide whether to accept the device.

Endpoints and variables measured

In this study, we classified patients as "acceptance" if they consented during the initial outpatient consultation and as "non-acceptance" if they refused. If neither acceptance nor non-acceptance was documented after the initial explanation, the patient was classified as non-acceptant. Whether patients accept isCGM is a dynamic process and may change over time. However, in this study, most patients who did not accept the device after the initial explanation consistently continued to decline it thereafter.

A binary outcome variable was defined based on whether the patient accepted or did not accept the use of isCGM, and logistic regression analysis was performed to identify explanatory variables influencing this decision. Patient-related explanatory variables included age, sex, total insulin dose, HbA1c level, duration of insulin use, body mass index (BMI), duration of diabetes, number of insulin injections per day, and frequency of SMBG. All parameters are as of the time isCGM was recommended. These variables were assessed in all patients by retrospectively reviewing their electronic medical records.

In the present study, other clinical data, such as the presence of hypertension, dyslipidemia, and medication profiles, were not included, as they were considered unlikely to have a major impact on the acceptance of isCGM. Data on educational level and income could not be obtained.

Statistical analyses​​​​​

Comparison Between Two Groups

Participants were divided into an isCGM acceptance group and an isCGM non-acceptance group, and the number of participants or median values for each factor were compared. Fisher's exact test was used for binary variables, and the Wilcoxon rank-sum test was used for continuous variables.

Data Formatting and Preparation

Data regarding total insulin dose, duration of diabetes, duration of insulin use, and HbA1c showed right-skewed distributions; therefore, a logarithmic transformation was applied to these variables. The frequency of SMBG showed a bimodal distribution; therefore, the values were divided into two groups, and each group was assigned its median value.

Excluding Highly Correlated Variables

To avoid multicollinearity, variables with high mutual correlation coefficients were excluded from the analysis. The frequency of insulin injections was excluded because it showed a relatively high correlation coefficient (r=0.67) with the total insulin dose. Eight variables were selected as independent variables: sex, age, HbA1c, BMI, SMBG frequency, duration of insulin use, duration of diabetes, and total insulin dose.

Logistic Regression Analysis

Univariate and multivariable logistic regression analyses were performed with CGM acceptance as the dependent variable. In the univariate analysis, each independent variable was entered separately to estimate crude ORs. In the multivariable analysis, all independent variables were entered simultaneously to estimate adjusted ORs.

This study is a hypothesis-generating study, and the factors known to influence acceptance of isCGM are not yet understood. An exploratory analysis was conducted to examine influential factors. For variable (8), the number of cases (142) and the number of events (44) were small. Furthermore, the eight independent variables showed weak but mutual correlations. 

Elastic net regression is a penalized regression method that is widely used in statistical learning. It reduces overfitting by adding a penalty term to the loss function [7]. Cross-validation is a technique that divides the data into a prespecified number of folds, fits the model using all but one fold, and evaluates the model on the remaining fold. This process is repeated so that each fold serves once as the validation set, and the results are then averaged. Cross-validation helps estimate out-of-sample performance and tune model parameters, thereby reducing overfitting [8].

Elastic net penalized logistic regression is useful for variable selection when the number of predictors is large relative to the sample size and when predictors are correlated. In addition, cross-validation can be used to reduce overfitting and to tune the penalty parameter. In this study, we used this method to identify important variables. We performed 10-fold cross-validation to determine the optimal value of λ, the tuning parameter for the elastic net penalty [7]. We referred to two commonly used values for model selection: λmin and λ1se. Here, λmin was defined as the value of λ that minimized the mean cross-validated binomial deviance, whereas λ1se was defined as the largest value of λ within 1 standard error of the minimum deviance. In general, selecting λmin yields the model with the best cross-validated fit, whereas selecting λ1se yields a more parsimonious model with fewer variables. Although the λ1se model may show slightly lower apparent fit, it is often preferred when model simplicity and robustness are prioritized [9].

Because variable selection can vary depending on cross-validation fold assignments, the elastic net procedure was repeated 200 times using different fold partitions. Selection frequency was calculated as the percentage of repetitions in which each predictor was selected. Bidirectional stepwise analysis was performed as a sensitivity analysis, and the results were compared. As this was an exploratory, hypothesis-generating study, no sample size calculation was performed. All p-values are nominal and should be interpreted accordingly; findings require confirmation in future hypothesis-testing studies.

Statistical analyses were performed using R Version 4.4.1 (R Foundation for Statistical Computing, Vienna, Austria). Generative artificial intelligence (AI) tools, namely, ChatGPT-5.4 (OpenAI, San Francisco, California, United States), Perplexity (Perplexity AI, Inc., San Francisco, California, United States), and Claude (Anthropic, San Francisco, California, United States), were used to assist in writing the R code. Analytical decisions and interpretation were performed by the authors, with AI used only as supportive assistance.

## Results

Clinical characteristics

Of the 142 individuals analyzed, 77 were male. The median age was 72 years, the median duration of diabetes was 19 years, the median duration of insulin use was five years, and the median BMI was 23 kg/m^2^. In total, 98 individuals (69%) agreed to the recommended use of isCGM (Table 1).

**Table 1:** Clinical characteristics of all the patients BMI: body mass index; HbA1c: hemoglobin A1c; SMBG: self-monitoring of blood glucose

Variable	N (%)/median (IQR)
Accept	98 (69%)
Male	77 (54.2%)
Age (years)	72 (61-77)
Injection frequency (times/day)	1 (1-3)
SMBG frequency (times/month)	30 (15.0-52.5)
HbA1c (%)	7.8 (7.3-8.7)
Total insulin dose (units)	12 (6-20)
Duration of diabetes (years)	19 (10-20)
Duration of insulin use (years)	5 (1-13)
BMI (kg/m^2^)	23.0 (20.0-26.9)

Comparison between groups

The comparison between the acceptance group and the non-acceptance group is shown in Table 2. Total insulin dose, duration of insulin use, and BMI were significantly higher in the non-acceptance group.

**Table 2:** Comparison between groups (n (%) or median (IQR)) *p<0.05 P-values were calculated using Fisher's exact test for categorical variables and the Wilcoxon rank-sum test for continuous variables. Two-sided p-values are shown. BMI: body mass index; HbA1c: hemoglobin A1c; SMBG: self-monitoring of blood glucose

Variable	Accept (n=98)	Non-accept (n=44)	P-value
Male (%)	55 (56.1%)	22 (50%)	0.59
Age (years)	73.0 (63.2-78.0)	70.5 (59.8-75.0)	0.17
Injection frequency (times/day)	1.0 (1.0-3.0)	2.0 (1.0-3.0)	0.08
SMBG frequency (times/month)	30.0 (15.0-30.0)	30.0 (15.0-60.0)	0.28
HbA1c (%)	8.1 (7.3-8.9)	7.7 (7.2-8.2)	0.1
Total insulin dose (units)	10.0 (6.0-16.0)	15.5 (8.0-26.0)	<0.01*
Duration of diabetes (years)	16.0 (8.5-20.0)	20.0 (13.8-20.0)	0.26
Duration of insulin use (years)	3 (1.0-11.5)	10 (5.0-15.0)	<0.01*
BMI (kg/m^2^)	22.3 (20.0-26.0)	25 (21.8-28.0)	0.03*

Univariate and multivariable logistic analyses

In the univariate logistic regression analysis, the logarithmic transformation of total daily insulin dose (log_TDD), duration of insulin use (log_insulin), and BMI were significantly associated with lower odds of accepting isCGM use, although no variables were significant in the multivariable logistic regression analysis. The effect of age was small, with a low and non-significant odds ratio (Tables 3-4).

**Table 3:** Crude OR for acceptance of isCGM estimated using logistic regression models Outcome: acceptance of isCGM (coded 1=accept and 0=non-accept) *p<0.05 isCGM: intermittently scanned continuous glucose monitoring; OR: odds ratio; BMI: body mass index; log_HbA1c: logarithmic transformation of hemoglobin A1c; SMBG: self-monitoring of blood glucose; log_TDD: logarithmic transformation of total daily insulin dose; log_diabetes: logarithmic transformation of duration of diabetes; log_insulin: logarithmic transformation of duration of insulin use

Variable	Crude OR (95% CI)	P-value
Male	1.28 (0.63-2.62)	0.5
Age	1.02 (0.99-1.05)	0.22
SMBG frequency	0.98 (0.96-1.01)	0.24
log_HbA1c	6.77 (0.65-87.3)	0.12
BMI	0.92 (0.84-0.99)	0.03*
log_TDD	0.46 (0.27-0.75)	<0.01*
log_diabetes	0.71 (0.43-1.09)	0.14
log_insulin	0.61 (0.43-0.84)	<0.01*

**Table 4:** Adjusted OR for acceptance of isCGM estimated using logistic regression models Outcome: acceptance of isCGM (coded 1=accept and 0=non-accept) *p<0.05 isCGM: intermittently scanned continuous glucose monitoring; OR: odds ratio; BMI: body mass index; log_HbA1c: logarithmic transformation of hemoglobin A1c; SMBG: self-monitoring of blood glucose; log_TDD: logarithmic transformation of total daily insulin dose; log_diabetes: logarithmic transformation of duration of diabetes; log_insulin: logarithmic transformation of duration of insulin use

Variable	Adjusted OR (95% CI)	P-value
Male	1.46 (0.68-3.17)	0.33
Age	1.01 (0.98-1.04)	0.59
SMBG frequency	0.99 (0.96-1.02)	0.57
log_HbA1c	7.73 (0.51-152)	0.16
BMI	0.93 (0.85-1.02)	0.15
log_TDD	0.66 (0.35-1.22)	0.19
log_diabetes	0.80 (0.42-1.46)	0.48
log_insulin	0.72 (0.45-1.10)	0.14

Penalized regression analysis

**Figure 1:**
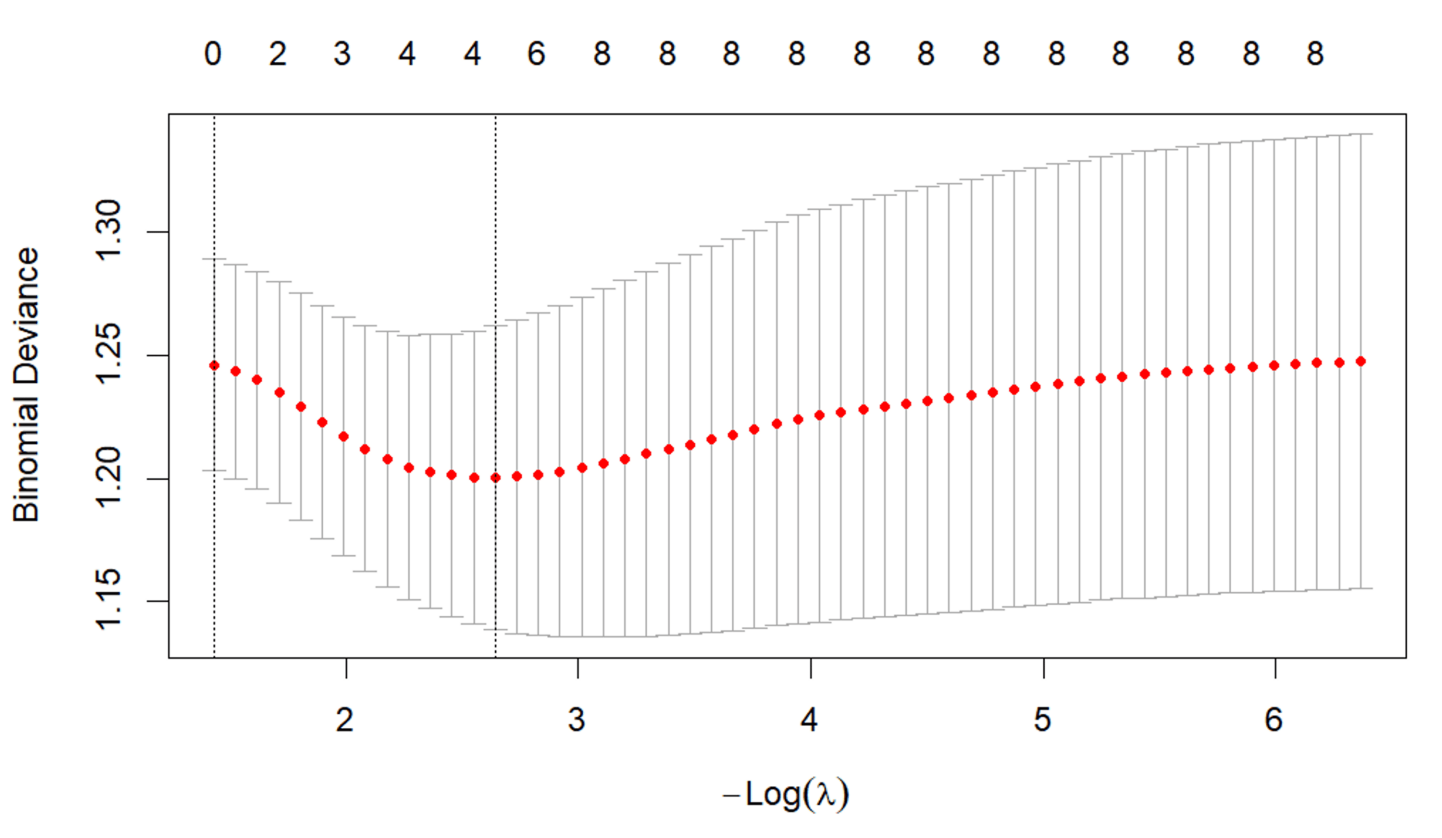
Cross-validation plot for the elastic net logistic regression model The red dots represent the mean cross-validated binomial deviance for each value of the penalty parameter λ, and the gray bars indicate the corresponding standard errors. The two vertical dotted lines indicate λ1se and λmin. λmin is the value of λ that minimizes the mean cross-validated binomial deviance, whereas λ1se is the largest value of λ within 1 standard error of the minimum deviance. The numbers shown at the top of the plot indicate the number of non-zero coefficients in the model at each value of λ.

In the elastic net logistic regression, 10-fold cross-validation was applied to tune the penalty parameter. At λ1se, no variables were retained, whereas at λmin, four variables (BMI, log_TDD, log_insulin, and log_HbA1c) were selected. The mean cross-validated deviance was 1.26 at λ1se​ and 1.19 at λmin, indicating only a modest improvement in predictive performance with the λmin model (Figure 1).

The AUC was 0.63, suggesting that the model had limited discriminative ability. The coefficients for BMI, log_TDD, and log_insulin were negative, whereas that for log_HbA1c was positive. In a stability analysis based on 200 repeated runs, BMI, log_TDD, and log_insulin were selected in 100% of runs, and log_HbA1c was selected in 98% of runs (Table 5).

**Table 5:** Selection frequencies of candidate variables for acceptance of isCGM across 200 repeated elastic net logistic regression analyses Outcome: acceptance of isCGM (coded 1=accept and 0=non-accept) In each repetition, λmin was selected by 10-fold cross-validation as the value of λ that minimized the mean cross-validated binomial deviance. Selection frequency indicates the proportion of repeated runs in which the coefficient was non-zero at λmin. Coefficient direction is reported only for variables with high selection frequency; directions were omitted for infrequently selected variables. Coefficient direction indicates the direction of association on the log-odds scale; negative coefficients correspond to lower odds (OR <1) and positive coefficients correspond to higher odds (OR >1). isCGM: intermittently scanned continuous glucose monitoring; BMI: body mass index; log_HbA1c: logarithmic transformation of hemoglobin A1c; SMBG: self-monitoring of blood glucose; log_TDD: logarithmic transformation of total daily insulin dose; log_diabetes: logarithmic transformation of duration of diabetes; log_insulin: logarithmic transformation of duration of insulin use

Variable	BMI	log_TDD	log_insulin	log_HbA1c	Age	Frequency of SMBG	log_diabetes	Male
Selection frequency (%)	100	100	100	98	36.5	18.0	5.5	4.5
Direction of coefficient (λmin)	Negative	Negative	Negative	Positive	-	-	-	-

Among predictors retained by elastic net at λ_min, log_TDD and log_insulin were moderately correlated (r=0.47), while other pairs showed low correlation, suggesting limited collinearity among selected variables. P-values and confidence intervals were not reported for the elastic net model because penalized regression is primarily used for variable selection and shrinkage, and conventional inferential statistics from ordinary logistic regression are not directly applicable after penalization and data-driven selection [7,10].

On the other hand, the bidirectional stepwise method selected three variables: BMI, log_TDD, and log_insulin (Tables 6-7). The results from the elastic net and variable selection via stepwise analysis were largely consistent. Specifically, insulin duration (log_insulin) and total insulin dose (log_TDD) were associated with reduced odds of accepting isCGM use. Higher log_HbA1c levels were associated with increased odds of accepting isCGM use. The effect of BMI was small.

**Table 6:** Crude OR for acceptance of isCGM estimated using logistic regression models after variable selection (bidirectional stepwise method) *p<0.05 Outcome: acceptance of isCGM (coded 1=accept and 0=non-accept) isCGM: intermittently scanned continuous glucose monitoring; OR: odds ratio; BMI: body mass index; log_insulin: logarithmic transformation of duration of insulin use; log_TDD: logarithmic transformation of total daily insulin dose

Variable	Crude OR (95% CI)	P-value
log_insulin	0.61 (0.43-0.84)	<0.01
log_TDD	0.46 (0.27-0.75)	0.01*
BMI	0.92 (0.84-0.99)	0.03*

**Table 7:** Adjusted OR for acceptance of isCGM estimated using logistic regression models after variable selection (bidirectional stepwise method) *p<0.05 Outcome: acceptance of isCGM (coded 1=accept and 0=non-accept) isCGM: intermittently scanned continuous glucose monitoring; OR: odds ratio; BMI: body mass index; log_insulin: logarithmic transformation of duration of insulin use; log_TDD: logarithmic transformation of total daily insulin dose

Variable	Adjusted OR (95% CI)	P-value
log_insulin	0.66 (0.45-0.96)	0.03*
log_TDD	0.65 (0.35-1.16)	0.14
BMI	0.92 (0.84-1.02)	0.13

## Discussion

Barriers to introducing new devices

Recent reports indicate that the following factors influence acceptance of new devices such as CGM: patient readiness, awareness of the benefits of initiating a new device, and access to the equipment.

Patient readiness refers to their motivation and preparedness to participate in treatment, representing their commitment to therapy and adherence. It is crucial that the patient has the will to manage their own blood glucose effectively. The patient's knowledge of diabetes and their motivation for treatment are important. Without the patient's own desire to achieve good blood glucose control, consent for new devices cannot be obtained.

The benefits of isCGM and the burdens associated with treatment are contradictory. Starting isCGM reduces the number of SMBG sessions requiring fingerstick blood sampling, lowers the risk of hypoglycemia, and improves safety and quality of life. This is thought to be influenced by the patient's sense of self-efficacy. On the other hand, the burden associated with treatment includes socioeconomic burdens and psychological/physical burdens. The economic burden is considered significant. The extent of insurance coverage and the size of the out-of-pocket expense affect the treatment burden. Concerns about the physical burden of wearing the sensor or on the body and skin sensitivity also play a role.

Access to the device, meaning that new devices are provided without issue and patients themselves can use them without difficulty, is crucial. Whether patients can master the new technology or feel comfortable using it also matters. Some may be unfamiliar with electronic devices, while others may face difficulties in actual use due to issues with their hands, fingertips, or vision. Furthermore, whether adequate support is available after starting treatment is also a key point.

Furthermore, when initiating treatment, particularly the introduction of insulin pumps for type 1 diabetes, starting early after diagnosis is associated with better maintenance of glycemic control [11-15].

Key findings of this study

We investigated whether sex, age, HbA1c, diabetes duration, insulin duration, total insulin dose, SMBG frequency, and BMI influence acceptance of isCGM. Few reports have explicitly discussed whether these factors affect acceptance of new devices, making our study exploratory. However, by examining clinical factors related to device acceptance from a perspective different from the aforementioned report, it is possible to gain new insights and alternative viewpoints.

To the best of our knowledge, this study is the first to analyze the characteristics of patients who accept or do not accept isCGM in a population largely comprising older individuals with type 2 diabetes. This exploratory study revealed the following findings: (a) A high total insulin dose, a long duration of insulin use, and a high number of insulin injections are associated with a tendency to non-acceptance of isCGM. (b) Higher HbA1c levels may be associated with a tendency to accept isCGM use. (c) In a population with type 2 diabetes and a high proportion of older individuals, high age may not markedly impact the acceptance of isCGM use. 

A high total insulin dose, a long duration of insulin use, and a high number of insulin injections

A large total insulin dose and a long duration of insulin use were associated with a tendency to non-acceptance of isCGM use. Total insulin dose was strongly correlated with the number of insulin injections (r=0.67), as noted earlier in this paper, and was also correlated with the duration of insulin therapy (r=0.47). Total insulin dose reflects insulin requirements and is influenced by body weight and insulin resistance. Total insulin dose showed weak correlations with SMBG frequency and duration of diabetes (r=0.20 and r=0.21, respectively). In this study, we collectively considered these factors to be indicators of high treatment intensity. High treatment intensity may be associated with a lower likelihood of adopting isCGM.

The reason why high treatment intensity was associated with non-acceptance of isCGM is unclear. Based on the recent review discussed above, these patient groups may have low motivation for treatment and a weak desire to achieve good blood glucose control. They may also be more concerned about the burden than the benefits. Regarding device access and usability, it is unlikely that these patients' access to devices is worse than that of the older patients.

These patients have a long treatment history, and diabetes management occupies a substantial part of their daily lives. Having become accustomed to long-term treatment, they may perceive that their current management approach has not caused any particular problems and, consequently, may be reluctant to accept a new treatment strategy. This may be related to the aforementioned report that introducing new devices in the early stages of diabetes is more advantageous. 

Healthcare providers tend to assume that patients will willingly accept recommended treatment options. However, patient acceptance of treatment recommendations by healthcare providers is not always favorable [16-18].

Patients with chronic illnesses may experience treatment burden or fatigue, which leads to poor treatment adherence [19]. This psychological burden associated with treatment may make it difficult to accept new treatment modalities [20,21]. Although treatment burden may be associated with non-acceptance of isCGM, this remains unclear because the present study did not directly assess patient burden.

Patients with higher HbA1c and acceptance of isCGM

In exploratory analysis, higher HbA1c tended to be associated with acceptance, although this was not statistically significant. Several individuals with diabetes understand that one of the primary goals of treatment is lowering HbA1c levels. During each outpatient visit, healthcare providers highlighted elevated HbA1c levels and emphasized the importance of reducing these levels. Recognizing that one's HbA1c level is high may reinforce the perception that the treatment is experiencing setbacks, which could, in turn, lead to a greater willingness to accept a new treatment device recommended by healthcare professionals.

Nevertheless, a relationship between reduced treatment adherence and poor glycemic control has been reported. Importantly, medication adherence may deteriorate because of complex treatment regimens, the financial burden of medical care, and patients' negative perceptions, ultimately resulting in poor glycemic control [22]. Although younger age is considered a contributing factor to reduced treatment adherence, the present analysis included a population with a high proportion of older adults. In a population comprising a high number of older individuals, poor glycemic control may influence the acceptance of new treatment strategies.

Older individuals and acceptance of isCGM

In general, older adults are considered to have more difficulty accepting new technologies. Reports from the Netherlands indicate the lower usage rate of isCGM among older adults [23]. We initially expected that older patients would be less likely to accept isCGM use.

Age did not impact acceptance of isCGM use. Conversely, factors such as prolonged duration of diabetes and insulin therapy were associated with the tendency to non-acceptance of isCGM use. Even among older individuals, there are cases in which the duration of diabetes or insulin therapy is relatively short, suggesting that age alone cannot be considered an underlying factor for non-accepting isCGM use.

Typically, older individuals are less adept at learning new things or operating new devices. A study using publicly available databases in the Netherlands demonstrated that, although the use of isCGM has increased annually, its utilization remains lower among older adults than among younger individuals [23]. The authors suggest that barriers to access and resistance to new technologies may be contributing factors to the observed delay. 

The following factors were considered to clarify why age did not influence the acceptance of isCGM in our study. Hand function declines with age owing to degenerative changes and reduced blood flow to the fingers [24,25]. Therefore, older individuals may find SMBG via fingertip puncture challenging. In daily clinical practice, we frequently encounter older patients complaining of an inability to perform SMBG effectively. Conversely, isCGM allows for the acquisition of glucose readings simply by scanning.

Under Japan's healthcare system, out-of-pocket expenses vary by age and income, but generally decrease with age. As a rule, those under 70 pay 30% of costs, those aged 70-74 pay 20%, and those aged 75 and over pay 10%. 

In the cases examined here, the median number of SMBG tests was 1 (Table 1). For example, using an exchange rate of 1 USD=147 JPY, if a patient performs one fingerstick test per day, the increase in out-of-pocket costs would be approximately $9.50 with a 30% copayment, while the increase for isCGM would be approximately $25.50. The difference is about $16, and while the patient's financial situation also plays a role, the out-of-pocket cost will increase to some extent. However, since out-of-pocket costs tend to decrease with age among the elderly, they may not be as concerned about the financial burden.

We believe that these factors explain why older age did not markedly impact acceptance of isCGM.

Limitations

We have discussed the reasons why patients do not accept isCGM, but we have not directly measured the causes of refusal. All of our analyses are speculative, and we cannot draw definitive conclusions regarding the causes of non-acceptance. Although we made statistical inferences regarding factors associated with non-acceptance, the model's discriminatory power is weak (AUC=0.63).

The manner in which isCGM is recommended likely has a substantial impact on whether patients accept it. In this study, all patients were outpatients examined by a single physician at a single institution; thus, there is a potential for bias due to site-specific and physician-specific factors. Furthermore, there is no guarantee that Dr. A's outpatients are not biased compared to the facility's overall diabetic patients. External validity and generalizability are not considered good, and further studies are needed to determine whether similar results apply to other patient populations.

When patients asked questions, Dr. A explained the financial burden associated with medical care; however, patients' awareness of treatment costs was not adequately assessed. The median frequency of SMBG among patients was 30 times per month or once per day (Table 1). As shown in the Discussion section, the financial burden is higher when using isCGM. Although there was no significant difference in SMBG frequency between the acceptance group and the non-acceptance group (Table 2), it is not possible to assess the extent to which the increased financial burden influenced the decision not to accept isCGM. Since data on patient income and financial status were not collected, these factors cannot be evaluated either.　　　　　　　　　　

This was a retrospective observational study. The number of measured variables was relatively large compared with the sample size, and residual issues of overfitting and multicollinearity may remain. While variables related to patients' insulin injections have been measured, the use of other diabetes medications and the presence of comorbidities such as complications, hypertension, and dyslipidemia have not been assessed. These factors may influence patients' financial burden and could serve as potential confounders.

In this study, patients who did not consent after the initial explanation were defined as non-acceptors. Most patients consistently did not accept subsequent recommendations. A few patients consented after multiple explanations; however, the treatment of this group has not been studied.

This study excluded patients lacking decision-making capacity. Under Japan's health insurance system, out-of-pocket costs for older patients are relatively low. These facts weaken the comparability and generalizability of one of our conclusions: that older patients may not necessarily decline treatment using new technologies.

## Conclusions

In this single-center retrospective study, high treatment intensity, reflected by a higher total insulin dose, longer duration of insulin use, and a greater number of insulin injections, was associated with non-acceptance of isCGM among patients with type 2 diabetes. For patients with poor HbA1c control, emphasizing the importance of improving glycemic control and introducing isCGM as one means to achieve this goal may be beneficial. Finally, even for older patients, it may be appropriate to actively recommend isCGM from the early stages of the disease when deemed necessary. All findings are exploratory and require confirmation in larger prospective studies.
